# Does Tinnitus Distress Depend on Age of Onset?

**DOI:** 10.1371/journal.pone.0027379

**Published:** 2011-11-18

**Authors:** Winfried Schlee, Tobias Kleinjung, Wolfgang Hiller, Gerhard Goebel, Iris-Tatjana Kolassa, Berthold Langguth

**Affiliations:** 1 Department of Clinical and Biological Psychology, University of Ulm, Ulm, Germany; 2 Department of Otorhinolaryngology, University of Zurich, Zurich, Switzerland; 3 Interdisciplinary Tinnitus Clinic, University of Regensburg, Regensburg, Germany; 4 Department of Clinical Psychology, University of Mainz, Regensburg, Germany; 5 Medical-Psychomatic Hospital, Schoen Clinic Roseneck, Prien, Germany; 6 Department of Psychiatry and Psychotherapy, University of Regensburg, Regensburg, Germany; University of Southern California, United States of America

## Abstract

**Objectives:**

Tinnitus is the perception of a sound in the absence of any physical source of it. About 5–15% of the population report hearing such a tinnitus and about 1–2% suffer from their tinnitus leading to anxiety, sleep disorders or depression. It is currently not completely understood why some people feel distressed by their tinnitus, while others don't. Several studies indicate that the amount of tinnitus distress is associated with many factors including comorbid anxiety, comorbid depression, personality, the psychosocial situation, the amount of the related hearing loss and the loudness of the tinnitus. Furthermore, theoretical considerations suggest an impact of the age at tinnitus onset influencing tinnitus distress.

**Methods:**

Based on a sample of 755 normal hearing tinnitus patients we tested this assumption. All participants answered a questionnaire on the amount of tinnitus distress together with a large variety of clinical and demographic data.

**Results:**

Patients with an earlier onset of tinnitus suffer significantly less than patients with an onset later in life. Furthermore, patients with a later onset of tinnitus describe their course of tinnitus distress as more abrupt and distressing right from the beginning.

**Conclusion:**

We argue that a decline of compensatory brain plasticity in older age accounts for this age-dependent tinnitus decompensation.

## Introduction

Tinnitus is the perception of sound in the absence of an auditory stimulus. Averaged over all age groups 5–15% of the western population experience some form of tinnitus. Many people can cope with chronic tinnitus, but about 1–2% of the population experience significant impairments in their quality of life due to their tinnitus.

The prevalence of chronic tinnitus increases with increasing age, peaking at 14.3% in people between 60 and 69 years of age [1]. The increase in tinnitus prevalence with age is at least partly explained by the fact that hearing loss is an important risk factor for tinnitus and hearing loss prevalence also increases with age [2].

Neuroplastic processes play a crucial role both in the generation of tinnitus [3] and in the amount of suffering [4]. Imaging studies reveal that neuroplastic changes in the central auditory system are generating the tinnitus percept [5] and that coactivation of nonauditory structures in the frontal cortex and the limbic system are involved in tinnitus related distress [6], .

Studies in animals and humans have shown that the mechanisms of cortical plasticity change over the lifetime with a tendency of decreased and less efficient neuroplastic potential as demonstrated by decreased induction and maintenance of long-term-potentiation (LTP) [8] and reduced long-term depression (LTD)-like effects with advancing age [9].

With these changes in the neuroplastic potential across the life span, age may not only have an influence on the incidence of tinnitus, but also on tinnitus related distress. A first hint for such a relation is given by a large epidemiological study demonstrating that people with bothersome tinnitus are elder than those with non-bothersome tinnitus (mean age 42 vs 38; p<0.001) [10]. Considering that tinnitus duration also influences its annoyance [11] we focused here especially on the role of tinnitus onset. In detail we hypothesized that the age of tinnitus onset may influence the perceived tinnitus related distress. More specifically, we assumed that early tinnitus onset is associated with less distress than later tinnitus onset.

## Methods

### Sample Description

The database of the German Tinnitus League [11], [12] provides a large sample of data from tinnitus patients with a broad age distribution to test this hypothesis. It was collected by a mail survey that was conducted among the members of the German Tinnitus League (Deutsche Tinnitus Liga, DTL). The questionnaires contained a large variety of clinical and demographic data and also included the Mini Tinnitus Questionnaire (Mini-TQ, [13]). Out of the database we selected 3′878 questionnaires where data about the following items were complete and valid: age at assessment, age at tinnitus onset, report of hearing impairment and all items of the Mini-Tinnitus Questionnaire. In this sample, the mean age was 56.1 years (SD 12.1), ranging from 16 to 95 years, 41.1% of the sample were women. Data were collected by the Psychological Institute of the University of Mainz and the Roseneck Center of Behavioral Medicine in Prien, Germany.

### Material

The data analysis is based on the questionnaire items asking for age at assessment, tinnitus duration, type of tinnitus onset (gradually versus abrupt), subjective hearing impairment, tinnitus distress after tinnitus onset and on the Mini-TQ total score. The questionnaire also assessed some more tinnitus-related variables, which are not subject of this data analysis [12]. All participants were informed that the storage of the data will be completely anonymized. The study was in accordance with the declaration of Helsinki and was approved by the ethical committee at the University of Regensburg. The ethics committee waived the need for consent from the participants for the analysis of the anonymized dataset.

The Mini-TQ is a short and psychometrically validated version of the Tinnitus Questionnaire 14,15, which assesses the tinnitus distress on a one-dimensional scale with scores ranging from 0 (no annoyance) to 24 (maximum annoyance).

### Statistical Analysis

The influence of the age of tinnitus onset on the perceived tinnitus distress was tested by means of regression analysis. In order to exclude the complex confounding role of hearing loss only subjects who reported no hearing impairment were included in this analysis. Statistical analysis was done using the statistical software package R (www.r-project.org, version 2.7.2).

## Results

### Prevalence of hearing impairment among tinnitus patients

**Figure 1:**
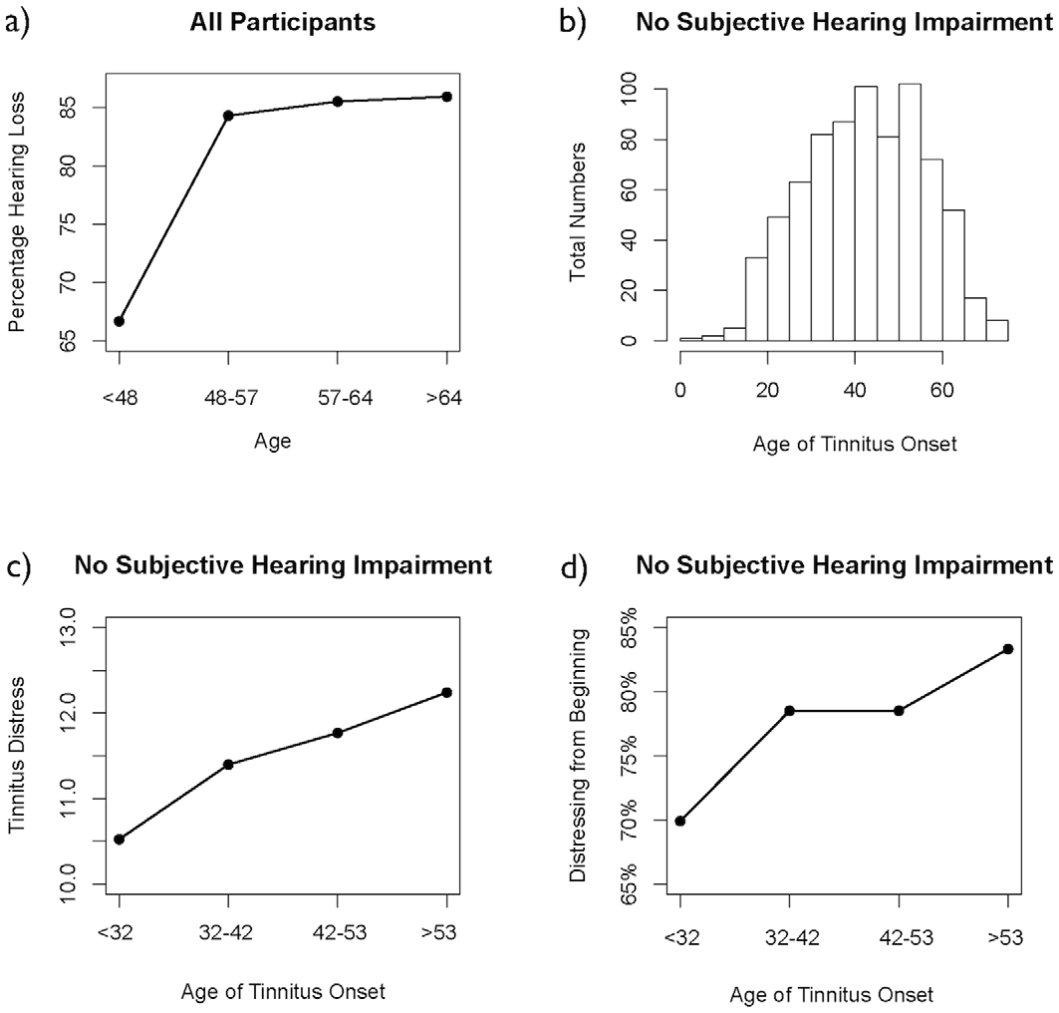
Graphical illustration of the sample data and results. a) The prevalence of hearing impairment increases with the participant's age. Prevalence data were aggregated with respect to age quantiles (Q1: 16–48 years, Q2: 48–57 years, Q3: 57–64 years, Q4: 64–95 years). Figures b) and c) report data from the group of subjects without subjectively reported hearing impairment. b) Histogram of the participant's age at tinnitus onset. c) Tinnitus distress increases with the age of tinnitus onset. The data were aggregated with respect to the age of onset quartiles (Q1: <32 years, Q2: 32–42 years, Q3: 42–53 years, Q4: 53–72 years). d) The percentage of patients that describe their tinnitus as “distressing from the beginning” increase for older age of onset. For illustration purposes, the sample was stratified as quartiles from early (1st quartile: tinnitus onset before the age of 32) to later tinnitus onset (2nd quartile: age 32–42; 3rd quartile: age 42–53; 4th quartile: over age 53). We found that the percentage of patients describing their tinnitus as distressing from the beginning augments with the tinnitus onset age 69.9 in the first quartile) up to 83.3% in the oldest quartile.

Overall, 80.5% of participants (3,123 out of 3,878) complained about some form of hearing impairment. The prevalence of subjective hearing impairment increased with the age of the tinnitus patients. In the lowest quartile of the sample (16–48 years), 66.7% patients reported an impairment of their hearing, which increased to 85.9% in the oldest quartile (64–95 years, see figure 1a).

### Age at tinnitus onset influences tinnitus distress

In the investigation of the relationship between tinnitus onset and tinnitus severity, hearing loss is an important confounding factor, which interacts in a complex way with age and tinnitus severity. First the prevalence of hearing loss increases with age, second hearing loss increases the risk for developing tinnitus [10], and third the amount of tinnitus distress is influenced by the distress the participant experiences because of the hearing loss accompanying the tinnitus. Thus, the following analysis concentrated on tinnitus patients that report a normal level of hearing (*n* = 755). Among those patients, the mean age of tinnitus onset was 42.4 years (SD = 13.5 years, see also the histogram in figure 1b). The mean total score of tinnitus distress according to the Mini-TQ was 11.5 (SD = 7.4). A stepwise regression analysis was calculated to investigate the association between the amount of tinnitus distress and the age at tinnitus onset. We started with a regression model explaining tinnitus distress by the age of the participant, the duration of tinnitus, the age at tinnitus onset, and the type of tinnitus onset (sudden onset vs. gradually increasing). Backward elimination based on Akaike's Information Criterion (AIC) was used to identify and exclude variables that do not improve the regression model significantly. Following this procedure, participant's age, duration of tinnitus, and type of tinnitus onset were eliminated and only the age at tinnitus onset remained in the model. In the final regression model, we found that tinnitus distress was associated with the age of the participant at the onset of the tinnitus (β = .05, t = 2.32, p = .02) demonstrating that patients with an earlier onset of tinnitus suffer less from their tinnitus (figure 1c).

Furthermore, a non-linear analysis was calculated and compared with the above described linear model. The comparison of the two models based on the AIC-criterion, however, revealed that the linear model is superior to the non-linear model suggesting a continuous increase of tinnitus distress with the participant's age at tinnitus onset.

### Age at onset of tinnitus influences the course of tinnitus distress

Additionally, the participants were asked if their tinnitus was distressing from the beginning or whether the tinnitus distress increased later on. Again, only participants without any subjective hearing impairment were included in the analysis. A logistic regression was calculated showing that the age at tinnitus onset predicts the probability that the tinnitus is perceived as stressful right from the beginning of tinnitus onset (z = 2.185, p = 0.029).

## Discussion

The main finding of our analysis is an influence of the age at tinnitus onset on tinnitus related distress. Higher age at tinnitus onset is associated with higher tinnitus related distress. To our knowledge this is the first report about an influence of age of tinnitus onset on tinnitus severity. This effect is independent from the age at tinnitus assessment, the duration of tinnitus and the type of tinnitus onset (gradual versus abrupt).

A large variety of different variables have been identified in the past as contributing factors to tinnitus distress, among them tinnitus loudness, hearing loss, vertigo/dizziness, hyperacusis, depression, anxiety and personality factors [11], [12], [16], [17]. Our study adds “age of onset” as an additional influencing factor underscoring the relevance of time related aspects in the pathophysiology of tinnitus. Earlier studies identified age and tinnitus duration as relevant factors. Age is strongly influencing tinnitus prevalence [1] and tinnitus duration plays an important role for response to treatment [18]–[21]. Though the effect of age of onset is statistically highly significant, it is rather small. However, considering the many variables, which exert a known influence on tinnitus related distress, this is not surprising.

Even though we cannot derive direct implications of our results on the clinical management of tinnitus patients, they may be relevant for a better understanding of both physiologic changes of brain function with increasing age and the pathophysiologic mechanisms involved in the generation of tinnitus distress. Many aspects of brain structure, brain function and brain plasticity are changing with age in a complex way [22]. These changes also involve adaptive and compensatory neural mechanisms [23]. Both the generation of tinnitus and the amount of tinnitus distress are thought to depend on adaptive and compensatory brain mechanisms [4]. In this context higher tinnitus distress at higher age of onset suggests an age-related decline in the efficiency of this compensatory mechanism for tinnitus. Thus our finding is in line with the observation that a decrease of cognition is related to higher tinnitus related distress [24]. Future studies are invited to further characterize the interactions between age related changes in neuroplastic potential, cognitive function and their influence on tinnitus distress.

Some limitations on the data analysis need to be noted here: The presented analysis is based on data from self report questionnaires of individuals with tinnitus and subjectively normal hearing. This selected sample may not be representative for all patients with tinnitus [25]. Also, the sample is restricted to active members of the German tinnitus patient association, which implies first a local selection and second a selection to people who are impaired by their tinnitus. Therefore, we are aware that our conclusions can only be preliminary as long as these data are not replicated in further independent samples. However, earlier analyses based on the same sample have provided results [8], [9], which are in line with the literature, indicating that the dataset is representative.

Tinnitus related distress is influenced by many variables such as tinnitus loudness, hearing loss, vertigo/dizziness, hyperacusis, depression, anxiety and personality factors. Here we suggest that the age at tinnitus onset might be an additional factor. Patients with a later onset of tinnitus in their life report greater distress than patients with an early tinnitus onset. The decline of neuroplasticity with advancing age might be an underlying mechanism for this observation.
